# Multi-dimensional dynamic assessment revealed the antibiotic-induced pseudo-germ-free model in SD rats stabilized within 4 days

**DOI:** 10.3389/fmicb.2026.1930662

**Published:** 2026-09-08

**Authors:** Yiduo Zhao, Lei Wang, Kang Zhang, Jingyan Zhang, Zhiting Guo, Xiaorong Lu, Jianxi Li

**Affiliations:** 1 College of Veterinary Medicine, Hebei Agricultural University, Baoding, Hebei, China; 2 Technical Innovation Center of Traditional Chinese Veterinary Medicine, Key Laboratory of Veterinary Pharmaceutical Discovery, Ministry of Agriculture and Rural Affairs, Lanzhou Institute of Animal Husbandry and Veterinary Medicine, Chinese Academy of Agricultural Sciences, Lanzhou, Gansu, China

**Keywords:** 3R principles, antibiotic treatment, fecal microbiota transplantation, pseudo-germ-free model, SD rats

## Abstract

Pseudo-germ-free (PGF) animal models are typically established after 7 days of antibiotic treatment, mostly validated using 16S rRNA sequencing. However, this method cannot distinguish live from dead bacteria and may overestimate the required depletion time. We investigated whether PGF model establishment in Sprague–Dawley (SD) rats could be completed in shorter than 7 days using an antibiotic cocktail. Gut microbiota was dynamically monitored with antibiotic cocktail treatment on days 0 to 7 by aerobic/anaerobic bacterial culture, Gram staining, and 16S rRNA sequencing, alongside body weight, food intake, and water intake. Body weight continued to increase in the Test group, while feed and water intake decreased only transiently during the first 2 days and returned to normal from day 3. No visible aerobic or anaerobic bacterial growth after 4 days and persisting through day 7. 16S rRNA sequencing showed that shared amplicon sequence variants (ASVs) with the Control group dropped from 379 on day 0 to 27 on day 4, stabilizing at 19–31 ASVs thereafter. Alpha diversity indices showed no significant changes after day 3 (*p* > 0.05). Beta diversity showed the peak period of microbiota restructuring on days 2–3 (*R*^2^ = 0.558, *p* = 0.001), while the effect size between adjacent time points dropped to an extremely low level after day 4 (*R*^2^ ≤ 0.025). LEfSe identified *Escherichia-Shigella* as the dominant genus. The multi-dimensional data demonstrated that 4 days of antibiotic cocktail treatment were sufficient to establish a stable PGF state in SD rats, aligning with 3R principles.

## 1 Introduction

As an important participant in host nutrient metabolism, immune regulation, and neuroendocrine control, the gut microbiota is crucial for host physiological health through the stability of its structure and function, and related functional research has become a frontier hotspot in the biomedical field (Adak and Khan, 2019; O’Riordan et al., 2025). In recent years, the proposal and development of the gut-X axis concept has provided an innovative perspective for elucidating the interaction mechanisms between the gut microbiota and multiple distal organs (Guo et al., 2026; Lin et al., 2025; Zhang et al., 2025, 2026). To clarify the causal relationship between gut microbiota and specific host phenotypes, the construction of PGF models has become indispensable, and PGF animal models have become essential research tools (Le Roy et al., 2019). Among them, the preparation of PGF animal models by depleting endogenous gut microbiota with broad-spectrum antibiotic mixtures has become a key model in FMT and causal mechanism studies because of its lower cost and operational convenience (Gheorghe et al., 2021).

However, there is still no unified standard for the duration of antibiotic cocktail treatment in PGF SD rat models, and 7–14 days are commonly used in PGF model studies (de Nies et al., 2022; Xiao et al., 2022; Saavedra et al., 2024). Studies have confirmed that antibiotic-mediated depletion of gut microbiota in mice can long-term interfere with apoptotic cell clearance pathways and is a potential inducer of inflammation and autoimmune dysregulation (Saavedra et al., 2024). Even under the shortest 7-day protocol, the daily gavage procedure itself can still induce sustained stress and interfere with experimental results, with particularly pronounced effects in behavioral and neuroendocrine-related assessments (Xiao et al., 2022). Therefore, shortening the modeling period is not only a pursuit of experimental efficiency, but also a measure to reduce confounding variables that interfere with causal inference. In terms of evaluation systems, most current studies rely on 16S rRNA gene sequencing technology (Emerson et al., 2017; Reikvam et al., 2011). Although this method can comprehensively analyze microbiota structure, it cannot distinguish between live and dead bacteria and thus may overestimate the actual persistence time of viable bacteria (Shao et al., 2023). By contrast, the agar culture method, as the gold standard in microbiology, can directly reflect the persistence of cultivable viable bacteria (Lagier et al., 2015).

Prolongation of the antibiotic cocktail treatment period not only affects experimental efficiency, but may also exert profound biological effects on recipient animals (Desai et al., 2016; Reikvam et al., 2011; Wlodarska et al., 2011; Kalghatgi et al., 2013; Moullan et al., 2015). That long-term antibiotic exposure can damage the integrity of the intestinal mucosal barrier and alter goblet cell number and mucus layer thickness, thereby affecting the colonization microenvironment for donor microbiota in subsequent FMT (Desai et al., 2016; Reikvam et al., 2011; Wlodarska et al., 2011). In addition, the direct inhibitory effect of antibiotics on mitochondrial function may persist for a long time after drug withdrawal, interfering with the baseline state of host energy metabolism (Kalghatgi et al., 2013; Moullan et al., 2015). More importantly, during the 7–14-day antibiotic treatment period, experimental animals undergo 7–14 gavage procedures, and this repetitive stress itself can alter intestinal permeability and the local immune microenvironment through sustained activation of the hypothalamic–pituitary–adrenal axis, making it difficult for investigators to distinguish the respective contributions of microbiota depletion and chronic stress to the experimental outcomes when evaluating FMT effects (Bailey et al., 2011; Mawdsley and Rampton, 2005).

In FMT experimental design, the duration of antibiotic depletion in recipients markedly affects transplant efficacy, as overly long depletion allows residual microbes to reoccupy gut niches and trigger colonization competition (Buffie et al., 2012; Dethlefsen and Relman, 2011; Ng et al., 2013). Therefore, clarifying when microbiota depletion reaches a steady-state plateau is decisive for FMT. However, current studies on antibiotic-induced microbiota depletion in SD rats report only the depletion effect at endpoint time points and lack refined continuous time-series characterization (Kennedy et al., 2018; Rakoff-Nahoum et al., 2015; Reikvam et al., 2011).

To address the above methodological gap and based on preliminary experiments, this study proposed the hypothesis that the establishment period of a PGF model can be significantly shorter than the traditional 7-day protocol and conducted experiments to verify it. Through parallel comparison of viable bacterial culture, Gram staining, and 16S rRNA sequencing of fecal samples, the dynamic depletion process of gut microbiota in SD rats receiving a combined antibiotic cocktail by daily gavage for 7 days was systematically monitored. These three methods cross-validated each other at different signal levels, effectively avoiding methodological bias caused by a single method. In addition, the design of daily continuous sampling allowed the kinetic characteristics of microbiota depletion to be fully presented, rather than being limited to endpoint judgment alone. This study aimed to determine the shortest duration of antibiotic treatment required for SD rats to reach a stable PGF state and to reveal the consistency and differences between viable bacterial culture and 16S rRNA sequencing in evaluating microbiota depletion.

## 2 Materials and methods

### 2.1 Experimental animals and ethics

Specific pathogen-free male Sprague–Dawley (SD) rats (5 weeks old, body weight 180 ± 20 g) were purchased from Chengdu GemPharmatech Co., Ltd. All rats were housed under standard controlled conditions (temperature: 22 ± 2 °C; 12 h light/dark cycle) with free access to standard laboratory chow and water. This study strictly adhered to the 3R principles (Replacement, Reduction, and Refinement) for animal experimentation and minimized the number of animals used while ensuring the reliability of the experimental data. At the same time, this study adopted a non-lethal experimental design; no animals were sacrificed for the purpose of the experiment during the study, and all animals were retained and continued to be housed after the end of the experiment.

### 2.2 Experimental design and antibiotic treatment

To ensure the reliability of the results, the entire experiment was repeated three times, with 16 rats used in each experiment. After 7 days of acclimatization, the rats were randomly assigned to two experimental groups: the Control group (*n* = 8) received normal saline and the Test group (*n* = 8) received a broad-spectrum antibiotic cocktail. Both groups were administered by gavage at a dose of 1 mL/kg body weight once daily for 7 consecutive days. All antibiotics were purchased from Beyotime Biotechnology (Shanghai, China). To ensure stable efficacy, the cocktail consisting of penicillin (200 mg/mL), metronidazole (200 mg/mL), neomycin (200 mg/mL), and vancomycin (100 mg/mL) was freshly prepared each day before use for gavage in the Test group (Le Roy et al., 2019; Qi et al., 2023). The actual daily doses administered were 200 mg/kg for penicillin, 200 mg/kg for metronidazole, 200 mg/kg for neomycin, and 100 mg/kg for vancomycin (Qi et al., 2023).

### 2.3 Sample collection

Fresh fecal samples from each rat were collected before treatment (Day 0) and daily after treatment (Day 1–Day 7). Samples were immediately used for bacterial culture and Gram staining after collection. The remaining samples were snap-frozen in liquid nitrogen, transferred and stored at −80 °C for subsequent 16S rRNA gene sequencing.

### 2.4 Aerobic bacterial culture

A 100 mg fecal sample from the middle portion was collected and homogenized in 1 mL PBS solution. After centrifugation at 100 ×*g* for 1 min, the supernatant was collected. The supernatant was diluted to 1 × 10–4 g/mL, and 5 μL was spotted onto Tryptic Soy Agar (TSA) blood plates and incubated aerobically at 37 °C for 48 h assessment of detectable bacterial growth of aerobic and facultatively anaerobic bacterial colonies.

### 2.5 Anaerobic bacterial culture

Under the same preparation conditions described above, 5 μL of the fecal supernatant was inoculated onto Schaedler blood agar plates, immediately transferred into an anaerobic jar, and incubated at 37 °C for 48 h assessment of detectable bacterial growth of obligate anaerobic bacteria.

### 2.6 Gram staining

A 100 mg middle-section fecal sample was collected and homogenized in 1 mL PBS solution. After centrifugation at 100 × g for 1 min, the supernatant was collected. The fecal supernatants from the 8 rats in each group were mixed thoroughly and diluted to 1 × 10^−3^ g/mL. A 20 μL aliquot was used for Gram staining. After drying and mounting, bacterial morphology was observed under oil immersion microscopy.

### 2.7 Monitoring of general animal condition

During the experiment, the mental status, water intake, food intake, and body weight changes of animals in each group were closely monitored and recorded daily to evaluate animal health status and the potential impact of the model construction process on physiological condition.

### 2.8 16S rRNA sequencing of rat fecal samples

#### 2.8.1 Sample selection

16S rRNA sequencing was performed on fecal samples from Rep-1.

#### 2.8.2 Library construction and sequencing

Total DNA was extracted from rat fecal samples using a DNA extraction kit (DP328, TIANGEN). PCR amplification was performed using the gut microbiota V3-V4 region-specific primers 341F (5’-CCTAYGGGRBGCASCAG-3′) and 805R (5’-GTGCCAGCMGCCGGGGTAA-3′) on a Bio-rad T100 gradient PCR instrument. The amplified products were subjected to magnetic bead purification and target band recovery using a Universal DNA Purification and Recovery Kit (DP214-03, TIANGEN). Subsequently, libraries were constructed using the NEBNext Ultra II DNA Library Prep Kit (E7645B, NEB), and finally, sequencing was performed on a DNBSEQ-G99 sequencer (MGI).

To exclude potential reagent-derived contamination, sterilized ddH_2_O was used as the negative control (NC) in place of template DNA during library preparation. Only batches in which the NC wells showed no visible bands and the NC raw reads were below 100 were processed for downstream analysis.

#### 2.8.3 Bioinformatics analysis

First, the raw sequencing data were demultiplexed based on barcode and primer sequences to obtain sample-specific data. Subsequently, FLASH (Version 1.2.11) was used to merge the sequences after removing barcodes and primers to obtain Raw reads. The Cutadapt software was then used to trim the reverse primer sequences. Next, fastp software (Version 0.23.1) was employed for strict quality filtering of Raw reads to obtain high-quality. Then, chimeric sequences were removed by aligning against the Silva 138.1 database to obtain Clean reads for subsequent analysis. For the obtained Clean reads, the DADA2 (with --p-trunc-len 0) was used for denoising to obtain the final ASVs. Finally, the classify-sklearn algorithm from QIIME 2 (v2025.4) was used to perform species annotation on each ASV using a pre-trained Naive Bayes classifier.

### 2.9 Statistical analysis

For body weight and alpha diversity, two-way ANOVA was used. Beta diversity was assessed by PERMANOVA based on Bray–Curtis distance matrices with 999 permutations. Differential genera were identified by LEfSe (LDA > 4), and Kruskal–Wallis tests were used for comparisons of individual bacterial taxa across multiple time points. All analyses were performed using GraphPad Prism 9.5, with *p* < 0.05 considered significant.

## 3 Results

### 3.1 *In vitro* culture of rat fecal microbiota

**Figure 1:**
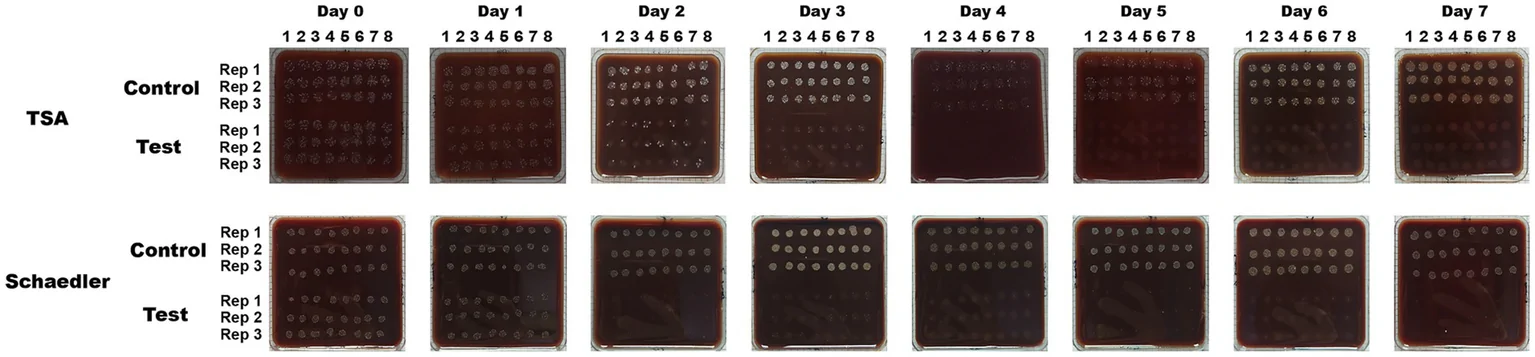
Combined antibiotic cocktail treatment reduces culturable aerobic and anaerobic fecal bacteria in SD rats starting (*n* = 8 per group).

As shown in Figure 1, under aerobic culture conditions, the number of culturable bacteria in the feces of the Control group remained stable at a relatively high level. In contrast, the number of aerobic bacteria in the feces of the Test group exhibited a daily decreasing trend, with no visible colony growth observed by day 4 (<2 × 10^6^ CFU/g). Under anaerobic culture conditions, we observed the same trend. Three replicate experiments yielded identical results.

### 3.2 Gram staining of rat feces

**Figure 2:**
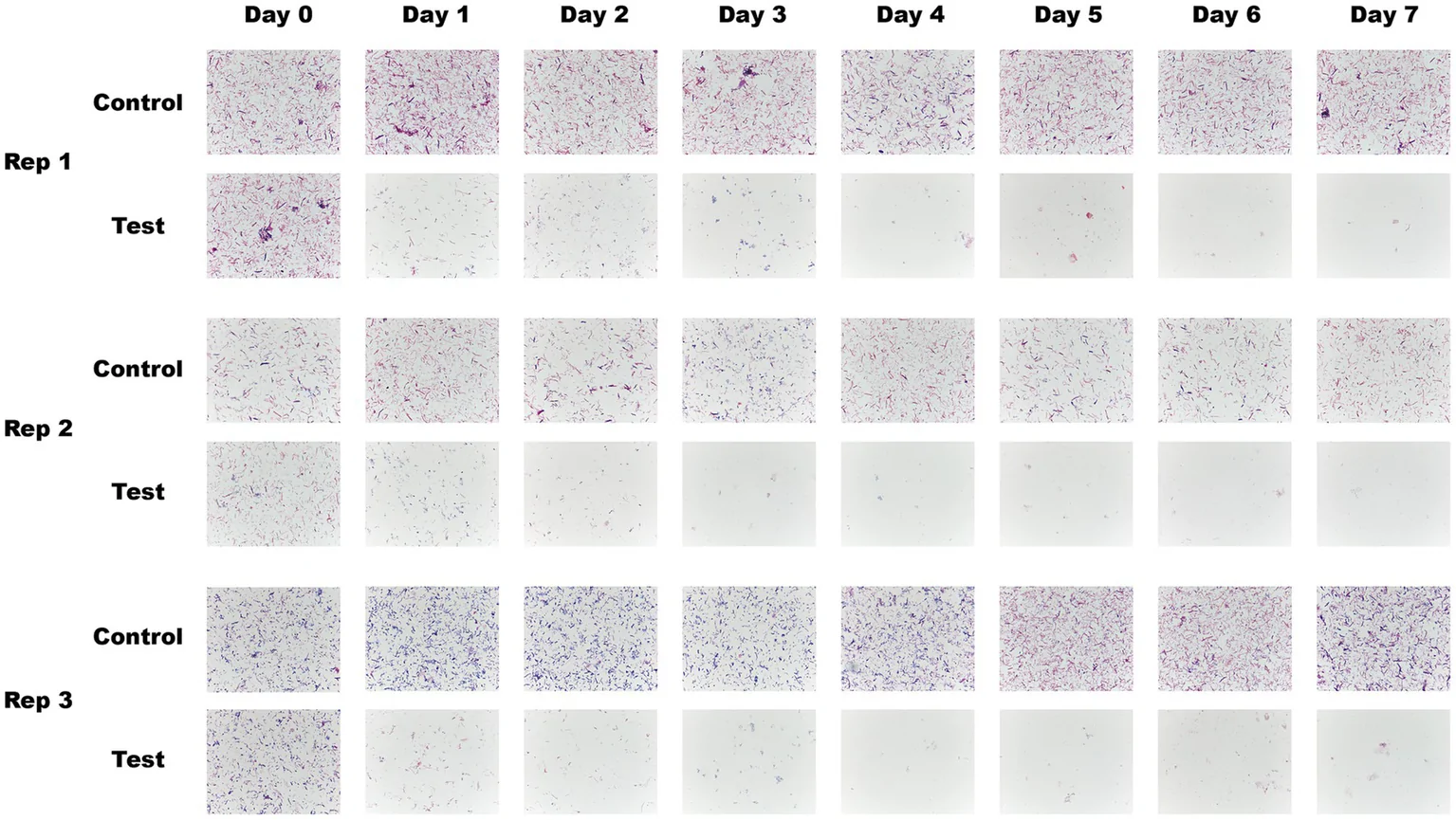
Combined antibiotic cocktail treatment reduces fecal bacterial diversity and intact bacterial morphology in SD rats over time (*n* = 8 per group).

As shown in Figure 2, the bacterial morphological diversity and abundance in the feces of the Control group remained stable at relatively high levels. In the Test group, bacterial diversity and abundance decreased over the experimental period. From day 4 onward, almost no intact bacterial morphology was observed in the microscopic field, and only a small amount of stained mucosal debris was visible.

### 3.3 Changes in rat body weight, water intake, and feed intake

**Figure 3:**
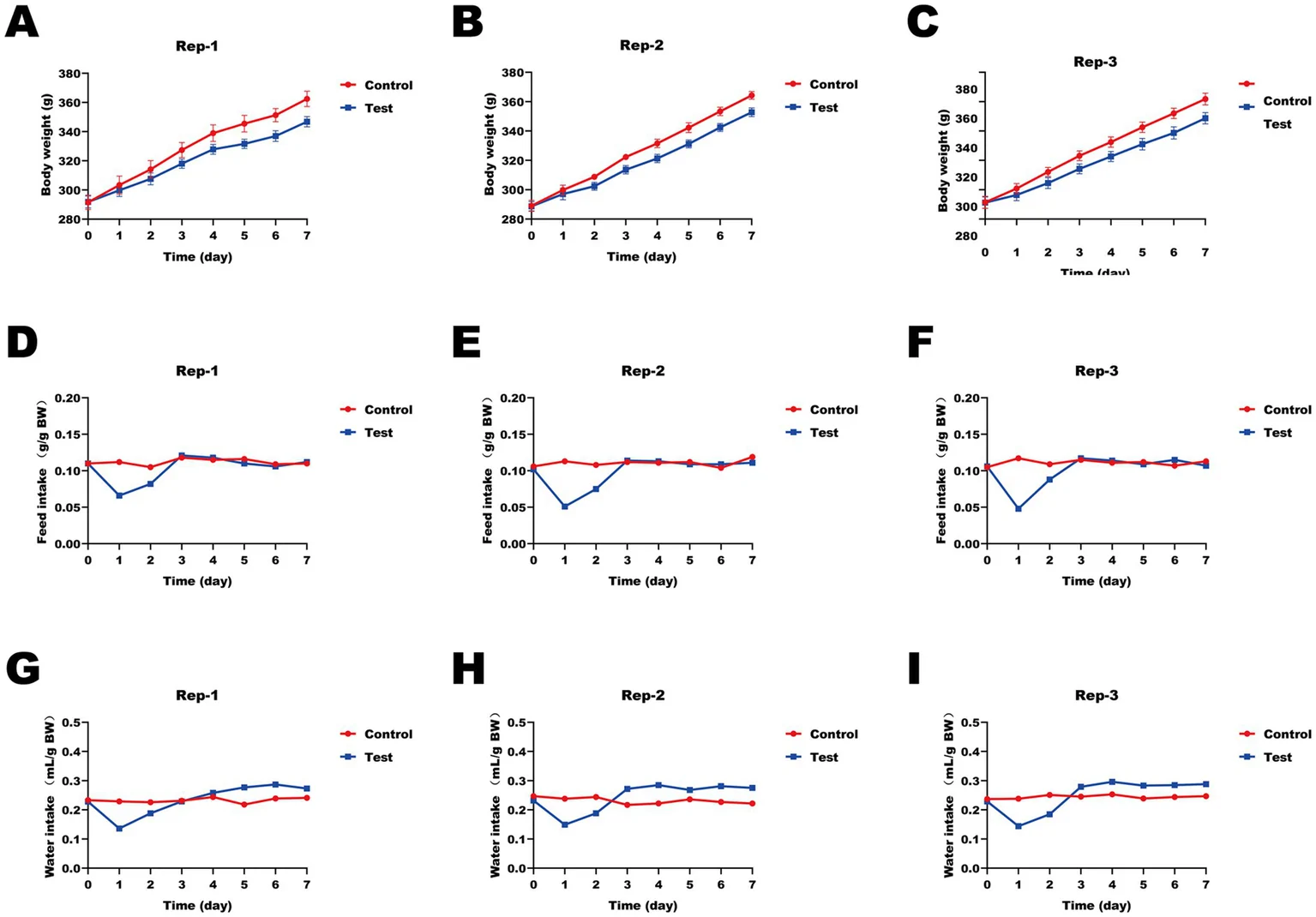
Changes in rat body weight (*n* = 8 per group). **(A)** Rep-1, **(B)** Rep-2, **(C)** Rep-3; changes in rat feed intake (FI). **(D)** Rep-1, **(E)** Rep-2, **(F)** Rep-3; changes in rat water intake (WI). **(G)** Rep-1, **(H)** Rep-2, **(I)** Rep-3.

Throughout the experiment, body weight continuously increased in both groups. The body weight gain in the Test group showed no significant difference compared to the Control group although it was numerically lower in the Test group (*p* > 0.05) (Figures 3A–C). The group × time interactionresults are as follows: Rep – 1: *F*_(7, 112)_ = 0.7314, *p* = 0.6457; Rep – 2: *F*_(7, 112)_ = 1.0190, *p* = 0.4219; Rep – 3: *F*_(7, 112)_ = 0.7439, *p* = 0.6353. The interaction effects among the three experimental groups were all non-significant (*p* > 0.05), consistently indicating that the weight gain trends in both groups of rats were parallel, and that antibiotic treatment did not significantly interfere with the temporal pattern of weight changes in the rats. During the first 2 days after the experiment started, feed intake (Figures 3D–F) and water intake (Figures 3G–I) decreased in both groups but returned to normal levels after day 3. The change trends were consistent across three replicate experiments.

### 3.4 Distribution of rat gut microbiota ASVs

Marker gene sequencing was performed on 128 fecal samples, generating a total of 13,629,559 raw reads, and the average number of reads per sample was 106,481 ± 4,616 (mean ± SD) (Supplementary Table S1). After quality trimming and removal of host genome contamination sequences, 11,739,215 clean reads were obtained, and the average of 91,713 ± 13,514 clean reads per sample (mean ± SD) (Supplementary Table S1). The ASV count statistics for each sample are displayed, the number and proportion of shared ASVs between the control and test groups gradually declined with prolonged antibiotic gavage (Supplementary Table S1). The NC samples yielded extremely low reads (≤55 raw reads), confirming that no significant contamination was introduced during library construction and sequencing (Supplementary Tables S2, S3).

**Figure 4:**
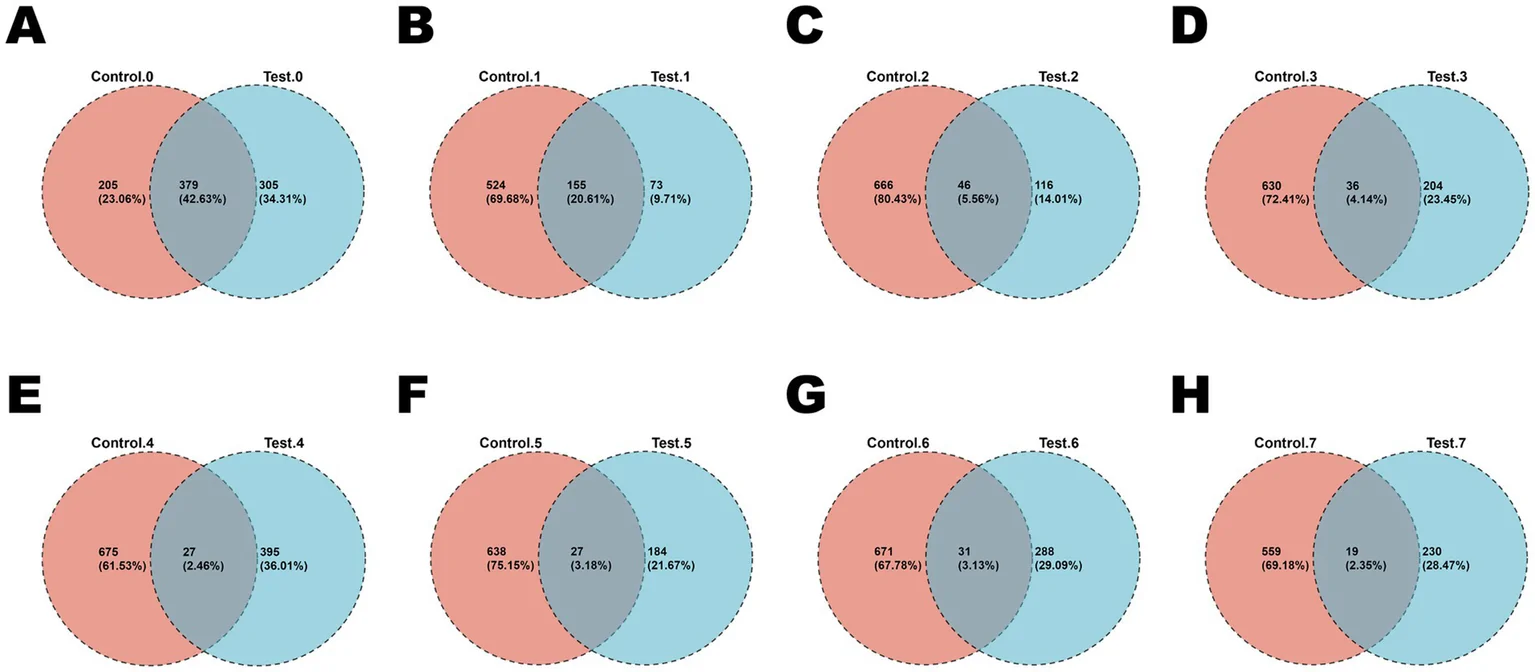
Shared ASVs between Control and Test groups at different time points after antibiotic gavage (*n* = 8 per group). **(A)** Day 0 (before administration), **(B)** Day 1, **(C)** Day 2, **(D)** Day 3, **(E)** Day 4, **(F)** Day 5, **(G)** Day 6, **(H)** Day 7.

Before antibiotic gavage, the Control and Test groups shared 379 ASVs, accounting for 42.63% (Figure 4A). After 1 day of antibiotic gavage, the Control and Test groups shared 155 ASVs, accounting for 20.61% (Figure 4B). After 2 days of antibiotic gavage, the Control and Test groups shared 46 ASVs, accounting for 5.56% (Figure 4C). After 3 days of antibiotic gavage, the Control and Test groups shared 36 ASVs, accounting for 4.14% (Figure 4D). After 4 days of antibiotic gavage, the Control and Test groups shared 27 ASVs, accounting for 2.46% (Figure 4E). After 5 days of antibiotic gavage, the Control and Test groups shared 27 ASVs, accounting for 3.18% (Figure 4F). After 6 days of antibiotic gavage, the Control and Test groups shared 31 ASVs, accounting for 3.13% (Figure 4G). After 7 days of antibiotic gavage, the Control and Test groups shared 19 ASVs, accounting for 2.35% (Figure 4H). These results indicate that antibiotics rapidly depleted the shared microbiota in the guts of both groups, with depletion reaching its lowest point on day 4, followed by a relatively stable plateau.

### 3.5 Analysis of rat gut microbiota alpha diversity

**Figure 5:**
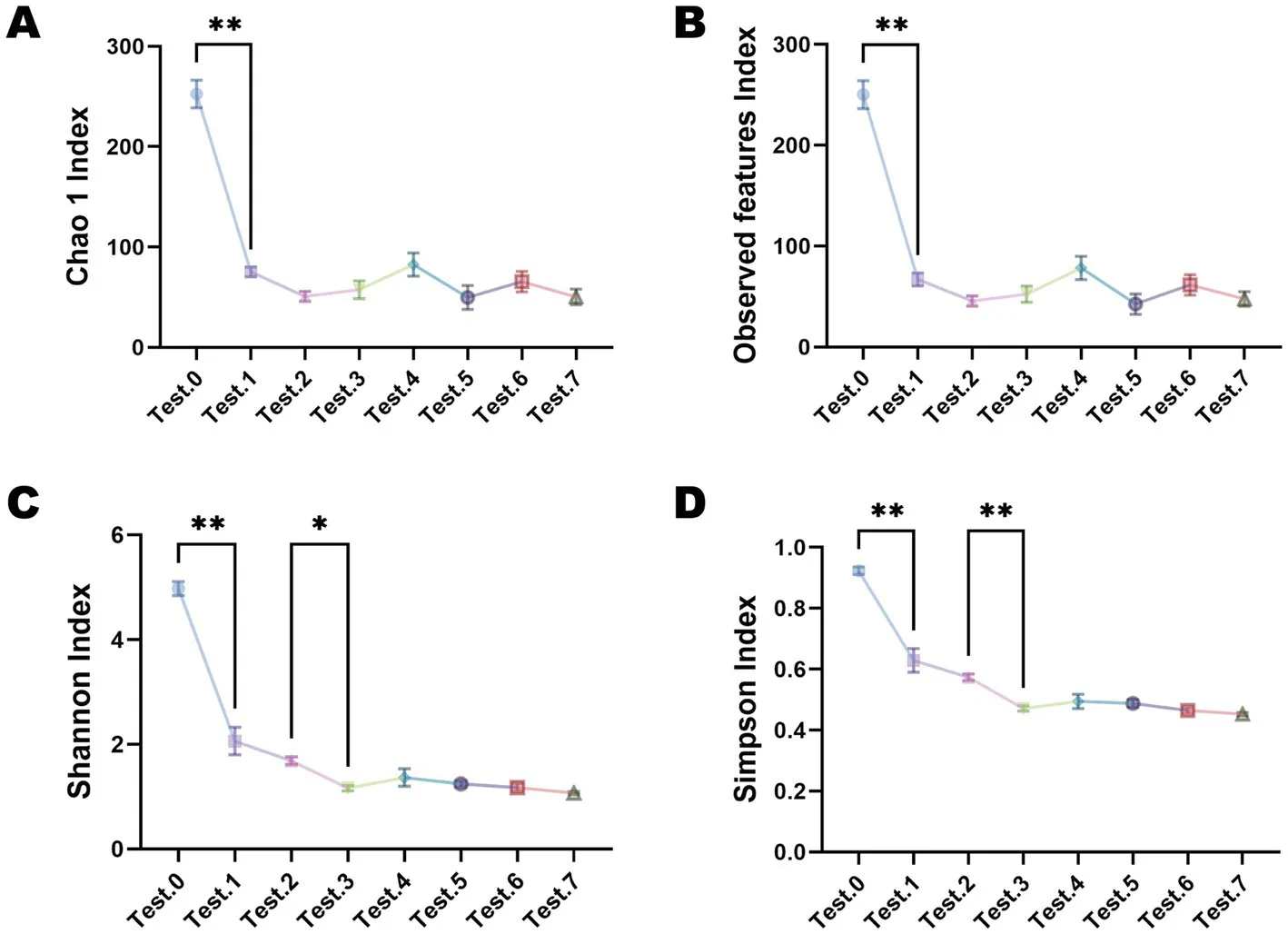
Comparison of gut microbiota alpha diversity among different time points following antibiotic gavage (*n* = 8 per group). **(A)** Chao 1 index. **(B)** Observed features index. **(C)** Shannon index. **(D)** Simpson index. ****: *p* < 0.0001, **: *p* < 0.01, *: *p* < 0.05.

To assess the impact of antibiotic treatment on the alpha diversity of the rat gut microbiota (Figure 5), we adopted a day-to-day adjacent comparison (Day n vs. Day n-1) strategy for each diversity index. The results showed that compared to Day 0, the Chao 1 index, Observed features index, Shannon index, and Simpson index all showed a highly significant decrease on Day 1 of antibiotic gavage (*p* < 0.05). In the adjacent comparison between Day 2 and Day 3, the Shannon index and Simpson index further decreased highly significantly (*p* < 0.05), while Chao 1 and Observed features showed no significant change during this period. From Day 4 of antibiotic gavage onwards, all indicators showed no significant differences in subsequent day-to-day comparisons.

### 3.6 Beta diversity analysis of rat gut microbiota

**Figure 6:**
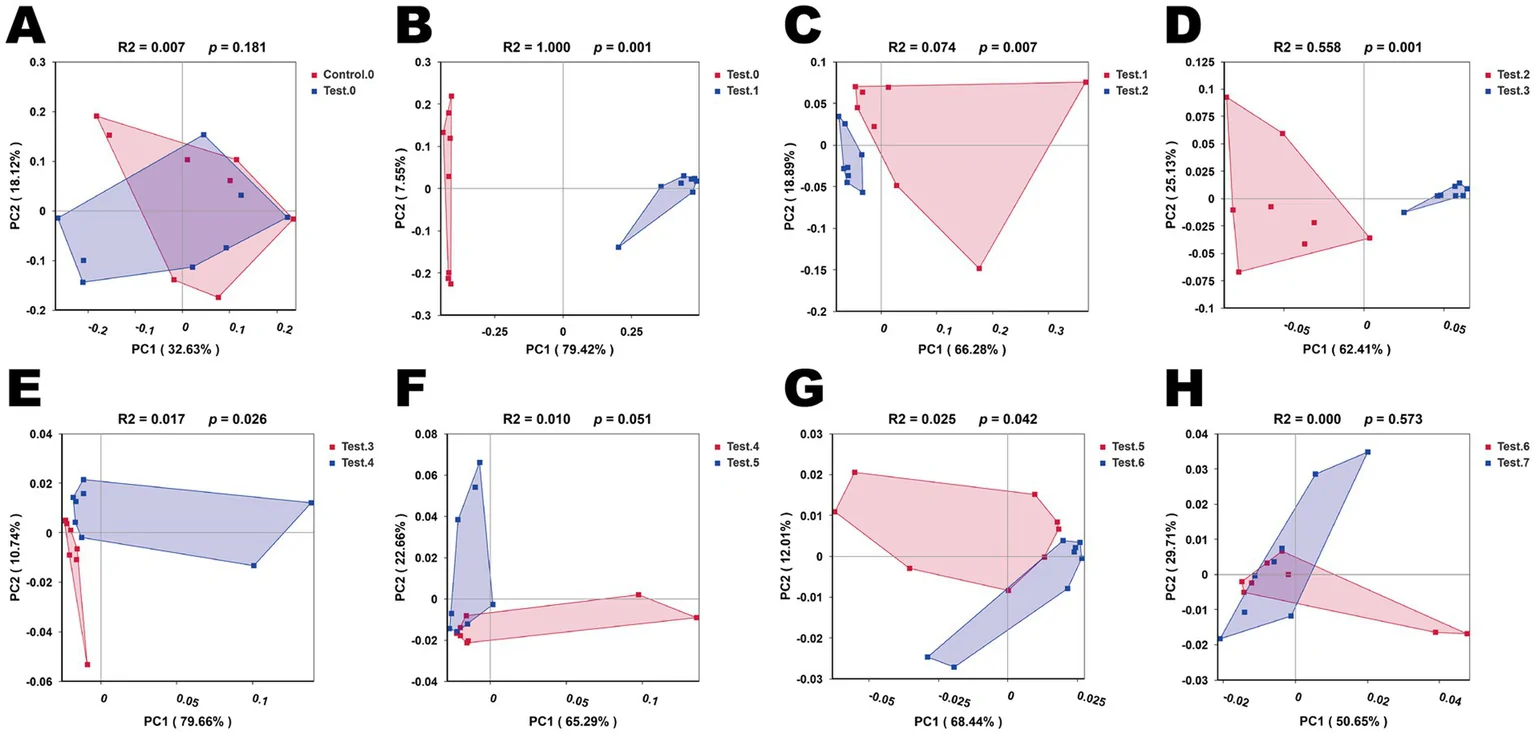
PCoA plots based on Bray-Curtis distance and pairwise PERMANOVA results showing dynamic shifts in rat gut microbial community structure after antibiotic cocktail gavage (*n* = 8 per group). **(A)** Control.0 vs. Test.0 (pre-treatment), **(B)** Test.0 vs. Test.1, **(C)** Test.1 vs. Test.2, **(D)** Test.2 vs. Test.3, **(E)** Test.3 vs. Test.4, **(F)** Test.4 vs. Test.5, **(G)** Test.5 vs. Test.6, **(H)** Test.6 vs. Test.7. *R*^2^ and *p* values represent the effect size and significance of PERMANOVA tests for community dissimilarity between paired groups; percentages on axes indicate the variance explained by each principal coordinate.

Pairwise PERMANOVA analysis and PCoA analysis based on Bray-Curtis distance revealed dynamic changes in gut microbiota structure at different time points after antibiotic treatment. Initially, the differences between Control.0 and Test.0 were not statistically significant and had a low effect size (*R*^2^ = 0.007, *p* = 0.181) (Figure 6A). Then, the inter-group difference between Test.0 and Test.1 showed the largest effect size and was highly significant (*R*^2^ = 1.000, *p* = 0.001) (Figure 6B). Following this drastic change, the differences between Test.1 and Test.2 were highly significant but had a relatively low effect size (*R*^2^ = 0.074, *p* = 0.007) (Figure 6C). Subsequently, a critical peak of microbial community restructuring was observed between Test.2 and Test.3 (*R*^2^ = 0.558, *p* = 0.001) (Figure 6D), where the effect size was tens of times greater than other adjacent time points, suggesting drastic community structure remodeling during this phase. From day 4 onwards, the effect sizes between adjacent time points dropped to low levels. The difference between Test.3 and Test.4 was significant but with a low effect size (*R*^2^ = 0.017, *p* = 0.026) (Figure 6E); the difference between Test.4 and Test.5 was not statistically significant and had a low effect size (*R*^2^ = 0.010, *p* = 0.051) (Figure 6F); the difference between Test.5 and Test.6 was significant but with a low effect size (*R*^2^ = 0.025, *p* = 0.042) (Figure 6G); and the difference between Test.6 and Test.7 was not statistically significant and had a low effect size (*R*^2^ = 0.0003, *p* = 0.573) (Figure 6H). These results indicate that the impact of antibiotic treatment on gut microbiota structure was mainly concentrated in the first 3 days, with the microbiota tending toward relative stability after day 4.

### 3.7 Distribution of rat gut microbiota after antibiotic treatment

**Figure 7:**
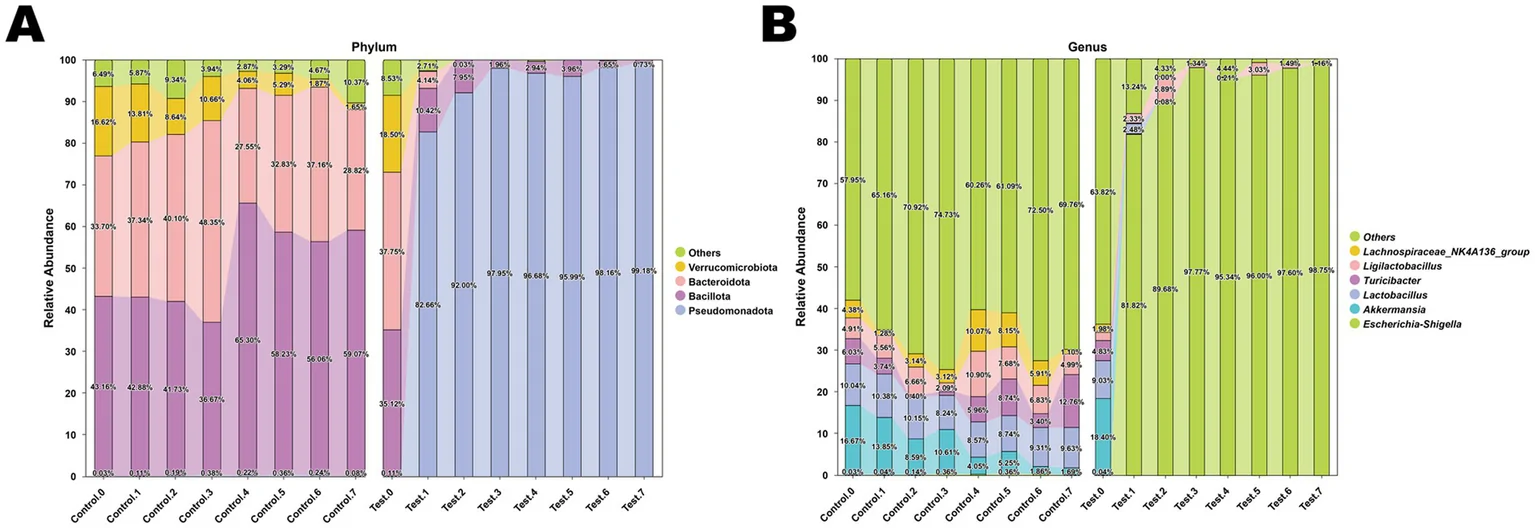
Shifts in the relative abundance of rat gut microbiota at the phylum and genus levels following antibiotic cocktail gavage (*n* = 8 per group). **(A)** Phylum-level microbial composition; **(B)** Genus-level microbial composition.

Antibiotic treatment led to drastic remodeling of the rat gut microbiota. At the phylum level (Figure 7A), the gut microbiota of normal rats (Control.0–Control.7 and Test.0) was mainly composed of *Bacillota*, *Bacteroidota*, and *Verrucomicrobiota*. After antibiotic treatment (Test.1–Test.7), the microbiota structure changed significantly, with the dominant phyla shifting to pseudomonadota and Bacillota. At the genus level (Figure 7B), the gut microbiota of normal rats was dominated by *Akkermansia*, *Lactobacillus*, and *Turicibacter*. After antibiotic treatment, *Escherichia–Shigella* became the absolute dominant genus.

### 3.8 Differential microbiota analysis after antibiotic treatment

**Figure 8:**
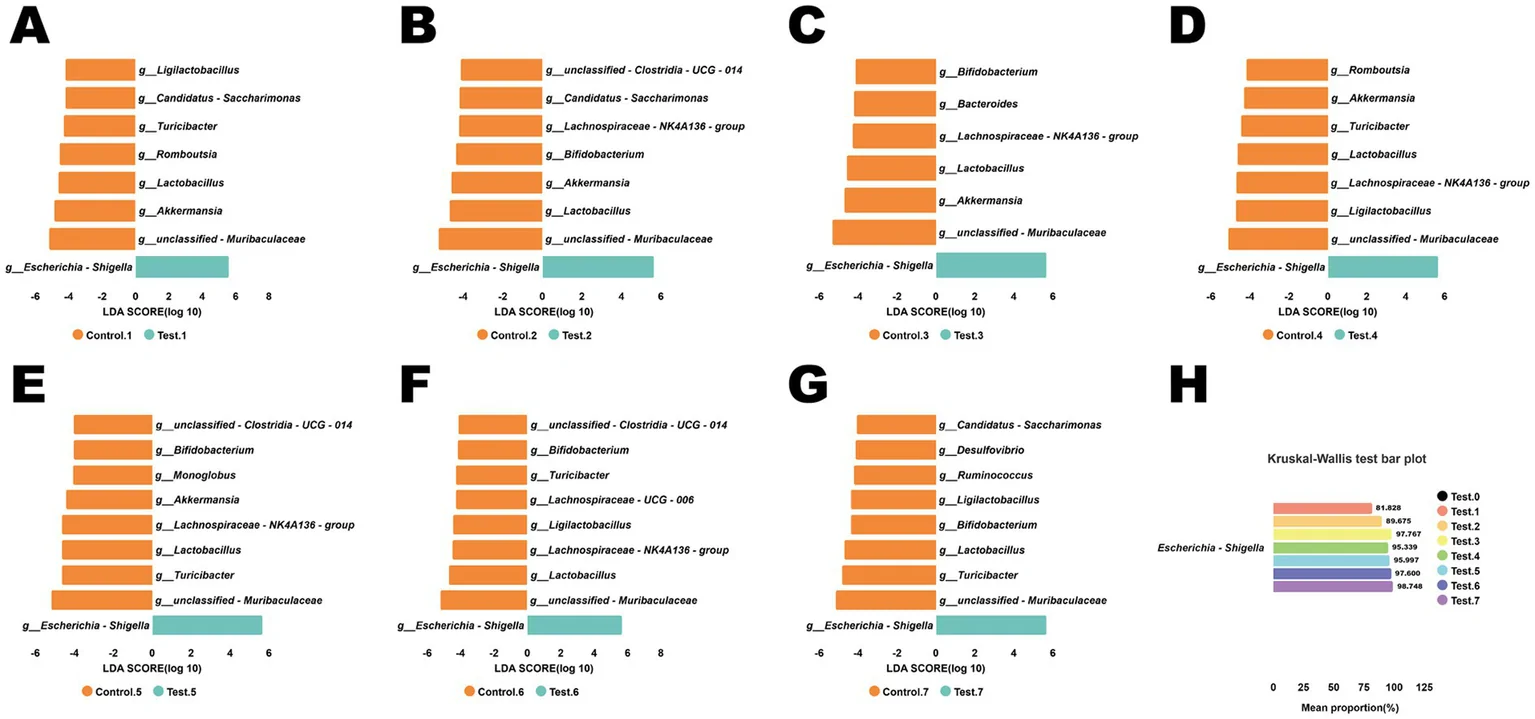
LEfSe linear discriminant analysis (LDA > 4) and Kruskal–Wallis test identifying differential gut bacterial genera between control and antibiotic-treated rats across 7 sampling days (*n* = 8 per group). **(A–G)** LEfSe LDA score plots comparing Control.n vs. Test.n at day 1 to day 7; **(H)** Kruskal–Wallis bar plot displaying the mean relative proportion of *Escherichia–Shigella* in the test group from day 0 to day 7.

LEfSe analysis (Figure 8) is shown when LDA > 4, the common differential genera in the Control group over the 7 days were *Lactobacillus* and *unclassified Muribaculaceae*. Additionally, *Akkermansia* was a common differential genus in the first 3 days, and *Turicibacter* was a common differential genus on days 4–7. From day 1 of antibiotic treatment, *Escherichia–Shigella* was the only differential genus in the Test group. Kruskal–Wallis rank sum test with multiple group comparisons showed that after 1 day of antibiotic treatment, the relative abundance of *Escherichia–Shigella* increased to 81.824%, reaching a peak of 97.767% after 3 days of treatment, followed by a plateau phase.

## 4 Discussion

This study systematically evaluated the depletion kinetics of a combined antibiotic cocktail on the gut microbiota of SD rats through parallel comparisons of viable bacteria culture, Gram staining, and 16S rRNA sequencing, validating the hypothesis that the cycle for establishing a PGF model can be significantly shorter than the traditional 7-day protocol. The results showed that after 4 days of antibiotic treatment, no bacterial growth was detectable under the culture conditions tested, and almost no intact bacterial were observed by Gram staining. Although 16S rRNA sequencing could still detect low levels of ASVs, the number of ASVs from day 4 to day 7 showed no significant difference (19–31 ASVs, accounting for 2.35–3.18%), indicating that the microbiota had entered a stable plateau phase. Absolute bacterial load was not quantified in this study, and thus 16S rRNA gene copies per gram of feces were not determined. However, cross-validation among the three methods consistently demonstrated that the gut microbiota of SD rats can reach a stable PGF state after 4 days of antibiotic treatment, with a clearance effect comparable to that achieved after 7 days. This finding provides a more operational and quantifiable time reference for the PGF model.

The methodological contribution of this study lies in incorporating viable bacteria survival, bacterial structural integrity, and bacterial DNA residues into the same evaluation system, comprehensively depicting the microbiota depletion process from three dimensions. Previous extensive studies have used 16S rRNA sequencing technology to determine the effectiveness of microbiota clearance, but DNA-level detection struggles to distinguish between live bacteria, dead bacteria, and free DNA (Cangelosi and Meschke, 2014; Carini et al., 2017; Emerson et al., 2017). This study found temporal differences in signal attenuation among the three methods. 16S rRNA sequencing could still persistently detect low-abundance ASVs from day 4 to day 7, whereas viable bacteria culture showed no detectable growth under these conditions after day 4, and Gram staining revealed almost no intact bacterial residues by day 4. Genus-level identification showed that the relative abundance of *Escherichia-Shigella* had exceeded 97%, indicating that the residual molecular signals almost entirely originated from a single genus. The concept of “relic DNA” proposed by Carini et al. suggests that DNA from dead microorganisms can significantly overestimate estimates of microbial diversity and community composition (Carini et al., 2017). Emerson et al. further summarized that nucleic acid-based detection results are not naturally equivalent to the presence of live bacteria, especially in complex microecological samples (Emerson et al., 2017). Studies by Young GR et al. found that bacterial DNA can persist in complex samples like feces for days to weeks after cell death and remain amplifiable for a certain period (Young et al., 2017). Therefore, the PCR amplification signal of dead bacterial DNA persists, forming a detectable molecular background noise, suggesting that previous reliance on 16S rRNA sequencing to assess microbiota clearance effectiveness overestimated the time required for antibiotic treatment. Hence, incorporating viable bacteria culture and Gram staining into the quality assessment system for PGF models helps to more accurately determine the endpoint of microbiota depletion.

Furthermore, during preliminary experiments, this study found that SD rats strongly resisted the mixed drinking water administration method, consistent with existing reports – BALB/c mice also refuse to drink antibiotic cocktail (Reikvam et al., 2011). Therefore, this study adopted daily gavage to ensure dosage accuracy. Although this operation increased experimental workload, it guaranteed the uniformity and reliability of antibiotic intake.

The dynamic changes in microbiota structure further support the above judgment. Beta diversity analysis showed that the most critical node was days 2–3 (*R*^2^ = 0.558, *p* = 0.001), where the effect size was tens of times higher than other adjacent time points, indicating extremely drastic community structure remodeling during this phase. Combined with changes in microbiota composition, this remodeling was manifested by a significant decrease in the relative abundance of *Akkermansia*, *Lactobacillus*, and *Turicibacter*, while *Escherichia-Shigella* proliferated rapidly and occupied the dominant ecological niche. Studies have confirmed that *Akkermansiaceae* and *Lactobacillaceae* can be completely absent after 7 days of antibiotic treatment, consistent with the dynamic observations of this study (de Nies et al., 2022). The second critical node was after day 4, where the effect size of comparisons between adjacent time points dropped to very low levels (*R*^2^ ≤ 0.025), and the community structure tended to stabilize. It appears that after undergoing rapid reorganization, the microbiota entered a relatively stable plateau phase starting from day 4.

LEfSe analysis further validated the above time points from the perspective of differential flora. In the Control group, *Lactobacillus* and *unclassified Muribaculaceae* were common differential genera over the 7 days, indicating they are important commensal members persistently present in the gut microbiota of normal SD rats and are most sensitive to antibiotic exposure. Studies have shown that *Lactobacillus* is a common commensal genus in the mammalian gut, playing important roles in maintaining intestinal barrier function, regulating local immunity, and inhibiting the proliferation of opportunistic pathogens (Shah et al., 2024; Walter, 2008). *Muribaculaceae* is one of the most abundant commensal families in the guts of mice and rats, closely related to complex polysaccharide utilization and host metabolic homeostasis (Lagkouvardos et al., 2019; Ormerod et al., 2016). *Akkermansia*, closely associated with mucin degradation, mucus layer renewal, and barrier homeostasis maintenance, was identified as a differential genus in the first 3 days (Cani and de Vos, 2017; Han et al., 2026). *Turicibacter*, related to host immune regulation and changes in the gut microenvironment, was identified as a differential genus on days 4–7 (Fung et al., 2019; Lynch et al., 2023). Their appearance at different time points suggests that the microbiota reorganization process is not unidirectional linear but involves complex dynamic interactions. In the Test group, from day 1 of antibiotic treatment, *Escherichia-Shigella* was identified as the sole persistent differential genus, maintaining this characteristic throughout the treatment period. Previous studies using combined antibiotic cocktails to construct PGF animal models have also observed the persistent enrichment of *Escherichia-Shigella*, which can become the dominant flora occupying a relatively high abundance after microbiota depletion (Ding et al., 2025; Ng et al., 2013; Theriot et al., 2014; Zarrinpar et al., 2018). This comparison further indicates that the microbiota structure of the Test group had completed its major remodeling process before day 4 following antibiotic treatment.

Antibiotic pretreatment is currently one of the most commonly used methods for constructing PGF FMT recipients (Kennedy et al., 2018; Reikvam et al., 2011; Zarrinpar et al., 2018). Combining findings from comparative reviews of existing FMT recipient models and related experimental studies, it is known that FMT should be performed as soon as possible after antibiotic discontinuation in PGF animals to minimize interference from the animal’s residual microbiota, thereby improving the colonization efficiency of the donor microbiota and the similarity between the recipient and donor microbiota (Buffie and Pamer, 2013; Kim et al., 2017; Ng et al., 2013; Safarian et al., 2016; Seekatz et al., 2014; Smillie et al., 2018). Therefore, for short-term mechanistic studies of the microbiota-gut-X axis, adopting a short modeling protocol avoids compensatory physiological changes caused by prolonged antibiotic treatment, allowing researchers to more precisely attribute effects to the absence of microbiota itself rather than the long-term exposure effects of antibiotics (Becattini et al., 2016; Cho et al., 2012; Langdon et al., 2016; Reikvam et al., 2011; Theriot et al., 2014; Zarrinpar et al., 2018). Our results indicate that FMT may be feasible as early as day 4, but this hypothesis needs to be verified by experiments comparing FMT on day 4 versus day 7.

Furthermore, the ethical advantages of the 4-day PGF model establishment method in terms of the 3R principles are also worth emphasizing. From a refinement perspective, the traditional 7–14 day protocol requires rats to undergo 7–14 gavage procedures, whereas shortening to 4 days requires only 4 gavage procedures. This can significantly reduce the stress and discomfort caused by repeated manipulation itself and shortens the continuous exposure time of antibiotics to the intestinal and systemic systems, reducing the probability of potential adverse reactions such as diarrhea, intestinal barrier damage, persistent microbial dysbiosis, and others (Becattini et al., 2016; Cho et al., 2012; Langdon et al., 2016; Reikvam et al., 2011; Theriot et al., 2014; Zarrinpar et al., 2018). Previous studies have also pointed out that antibiotic-induced gut microbiota imbalance may promote tumor metastasis, highlighting the unintended effects of long-term antibiotic exposure far beyond the gut itself, further emphasizing the necessity of shortening the modeling period (Iida et al., 2013; Li et al., 2024; Viaud et al., 2013). From a reduction perspective, a shorter modeling cycle reduces the risk of animals being excluded during the experiment due to complications related to procedures like gavage aspiration or intestinal injury, thus decreasing the need for additional animal numbers (Tannenbaum and Bennett, 2015; Turner et al., 2011). By reducing suffering, shortening exposure, stabilizing the model, and lowering procedural risks, this protocol better aligns the design of current animal experiments with the “Refinement” and “Reduction” dimensions of the 3R principles.

## 5 Conclusion

In summary, through parallel evaluation using viable bacteria culture, Gram staining, and 16S rRNA sequencing, demonstrated that continuous treatment with a combined antibiotic cocktail for 4 days can reduce the gut microbiota of SD rats to a stable low level, meeting the requirements for constructing a PGF recipient model. Compared with relying solely on 16S rRNA sequencing, a multi-method joint evaluation can more accurately identify the endpoint of microbiota depletion and reduce bias caused by residual bacterial DNA signals. This protocol shortens the experimental period while ensuring model establishment effectiveness and reduces procedural stress and antibiotic exposure, providing a more efficient strategy for constructing SD rat recipient models for microbiota-related research.

## Data Availability

The 16S rRNA sequences of rat fecal samples have been deposited into the NCBI Sequence Read Archive (SRA) under the accession number PRJNA1512181 (https://www.ncbi.nlm.nih.gov/sra/PRJNA1512181).
